# A New Year’s Message 2022

**DOI:** 10.3390/polym14030500

**Published:** 2022-01-27

**Authors:** Shin-ichi Yusa

**Affiliations:** Department of Applied Chemistry, Graduate School of Engineering, University of Hyogo, Himeji 671-2280, Japan; yusa@eng.u-hyogo.ac.jp

We greatly appreciate the contribution of Editorial Board Members, Guest Editors, reviewers, authors, and all related colleagues over the past year.

Unfortunately, the pandemic has not gone away in most countries and regions. However, the number of papers submitted yearly to *Polymers* in 2021 increased compared with those in 2020. We never give up on research in any difficult situation. This editorial deals with the most cited papers published in the year 2021 in the section “Polymer Chemistry” of the journal *Polymers*.

Simona et al. [1] reported edible packing from orange essential oil and trehalose in a carrageenan matrix. The produced films can act as a UV protector, and show antibacterial properties against Gram-negative bacteria and yeasts. This research is important information for further research and the possible practical application of these edible matrices. Atanase [2] reviewed the last decade concerning the preparation as well as the colloidal and biological characterization of micellar drug delivery systems based on natural biopolymers. Orellana et al. [3] reviewed the comprehensive analysis of conductive polymer nanocomposites presenting reversible dynamic bonds and their energetical activation to perform self-healing through the Joule effect. Mateos-Maroto et al. [4] reviewed an analysis of some of the current trends on the fabrication of Layer-by-Layer (LbL) materials using soft colloidal nano-surfaces, including liposomes, emulsion droplets, or even cells, as templates. Ali et al. [5] reported the kinetics and mechanism of ethylene and propylene homopolymerizations with ansa-zirconocenes activated by alkylaluminum/borate cocatalysts. Siddika et al. [6] reviewed a wide range of parameters for durability properties and challenges associated with waste glass concrete, which provides necessary guidelines for best practice with future research directions. Sánchez-Ruiz et al. [7] reviewed selected fully conjugated oligomers, dendrimers, and polymers, and briefly summarized their synthetic routes, aggregation-induced emission (AIE) properties, and potential applications. Dardano et al. [8] reported one-shot fabrication of polyethylene glycol diacrylate based hollow microneedles. In this case, any etching step is avoided, and the inner cavity of the hollow microneedles is fabricated at the same time as the external structure of the needles. Górski et al. [9] reported a new type of geopolymer as a promising material and suggested a new possibility of recycling cathode ray tube (CRT) glass without any special pretreatment. According to the mechanical performance, this metakaolin-based geopolymer with CRT glass in the form of an aggregate can be treated as a building material with excellent properties. Aranaz et al. [10] reviewed the state of the art of chitosan science, covering different aspects such as polymer chemistry, biological, and technological properties and applications in drug delivery and as a biocatalyst. Mrówka et al. [11] provided the effectiveness of using zinc ashes as abrasion-reducing fillers. The research also proved that reusing metallurgical production waste products is possible in polymer materials and has a positive impact on environmental protection. Le et al. [12] presented the mechanical properties of the hybrid composite thin-plates of the short basalt fibers/carbon textile-reinforced geomortar. Nappi et al. [13] reviewed the historic development and technologic challenges of stents, from first-generation bare-metal stents to newer devices. Milenin et al. [14] reported the confirmation of the presence of a specific interaction between high-generation carbosilane dendrimers in bulk, which manifested itself in a jump of the melt viscosity with an increasing dendrimer generation and an effective liquid–solid transition. Marx et al. [15] reviewed expanding monomers with a focus on the most recent activities and the elucidation of structure-property relationships, and also provided measuring techniques for the quantification of volumetric changes. Toledano-Osorio et al. [16] demonstrated that doxycycline-doped membranes were a potential candidate for use in guided bone regeneration procedures in several challenging pathologies, including periodontal disease. Bellotti et al. [17] reviewed the basic knowledge of photoinduced electron/energy transfer reversible addition–fragmentation chain-transfer (PET-RAFT) polymerization and explored the new possibilities that this innovative technique offers in terms of industrial applications, new materials production, and green conditions. Paolillo et al. [18] reviewed the intrinsic self-healing epoxy systems found in the literature, both as a standalone material and as matrices in polymer matrix composites, with an emphasis on those systems that are particularly suitable for aerospace applications. Ritzen et al. [19] reviewed a protocol for fused deposition modeling printing, and the testing of self-healing polymers was introduced using a previously reported self-healing thermoplastic polyurethane. Nenna et al. [20] reviewed the current evidence about polymers and nanoparticles for statin delivery in the field of cardiovascular disease.

In 2021, the following Special Issues in the section “Polymer Chemistry” were successful: “Polymers Synthesis and Characterization” edited by Edina Rusen, “Advanced Polymer Based Materials: Production, Characterization and Applications” edited by Roberto Avolio, Veronica Ambrogi, Rachele Castaldo, Giovanna Gomez D’Ayala, and Francesca De Falco, “Cross-linked Polymers” edited by Łukasz Klapiszewski and Beata Podkościelna, “Polymer Micelles II” edited by Shin-ichi Yusa, Pratap Bahadur, Hideki Matsuoka, and Takahiro Sato, “Advanced Polymeric Functional Materials Using Reversible Deactivation Radical Polymerization Techniques” edited by Ana Barros-Timmons, Elodie Bourgeat-Lami, and Amilton Martins dos Santos. The deadlines of the Special Issues of “Polymers Synthesis and Characterization” and “Cross-linked Polymers” are 30 April 2022 and 31 May 2022, respectively. Please submit your paper to these Special Issues.

There are signs of improvement against the pandemic, with the utilization of vaccines and drugs. Certainly, polymer chemistry can dissolve many problems of the modern era such as energy, environment, food, health, and so on. We hope 2022 will be a fruitful year.
